# CART (Cocaine- and Amphetamine-Regulated Transcript): A New Identified Intrafollicular Mediator in Ovulation Induction Protocols

**DOI:** 10.3390/biomedicines12112598

**Published:** 2024-11-13

**Authors:** Charalampos Voros, Despoina Mavrogianni, Sofoklis Stavros, Myrto Papamentzelopoulou, Evangelia Dimitroulia, Dimitrios Doumplis, Dimitris Mathiopoulos, Dimitrios Loutradis

**Affiliations:** 1 1st Department of Obstetrics and Gynecology, ‘Alexandra’ General Hospital, National and Kapodistrian University of Athens, 80 Vasilissis Sofias Avenue, 11528 Athens, Greece; depy.mavrogianni@yahoo.com (D.M.); mpntua@yahoo.gr (M.P.); 2 3rd Department of Obstetrics and Gynecology, Attikon Hospital, National and Kapodistrian University of Athens, Rimini 1, 12462 Chaidari, Greece; sfstavrou@yahoo.com; 3 Department of Microbiology, Medical School, National and Kapodistrian University of Athens, 75 Mikras Asias Street, 11527 Athens, Greece; e.dimitroulia@dunant.gr; 4 Fertility Institute-Assisted Reproduction Unit, Paster 15, 11528 Athens, Greece; ddoumplis@yahoo.com (D.D.); dimitrismathiopoulos@gmail.com (D.M.); loutradi@otenet.gr (D.L.); 5 Athens Medical School, National and Kapodistrian University of Athens, 15772 Athens, Greece

**Keywords:** infertility, gonadotropines, CART peptide, leptin, FSHR polymorphism, stimulation protocols

## Abstract

Background/Objectives: This study investigates the relationship between cocaine- and amphetamine-regulated transcript (CART) expression, leptin, and hormone profiles—specifically progesterone, testosterone, androstenedione, estradiol, follicle-stimulating hormone (FSH), and human chorionic gonadotropin (hCG)—across four distinct ovulation induction protocols (HMG, HMG/hCG, rFSH, and rFSH/hCG). It also investigates the relationship between follicle-stimulating hormone receptor (FSHR) Ser680Asn polymorphisms, CART expression, and in vitro fertilization (IVF) results, with the goal of better understanding how CART and FSHR polymorphisms affect ovarian response and oocyte quality. Methods: Data were obtained from 94 women who underwent controlled ovarian stimulation (COS) as part of their IVF therapy. Hormone levels, CART expression, and FSHR polymorphisms were measured across all four ovulation induction procedures. Statistical studies were undertaken to investigate the relationships between CART expression, hormone levels, and IVF results. Results: The study found no significant difference in body mass index (BMI) amongst the four stimulation procedures (*p*-values varied from 0.244 to 0.909). CART expression did not show a significant correlation with hormone levels throughout the whole cohort (progesterone, testosterone, androstenedione, FSH, hCG, and estradiol; *p* > 0.05). However, CART levels were adversely linked with the number of follicles > 12 mm (r = −0.251, *p* = 0.018), total oocyte count (r = −0.247, *p* = 0.019), and oocyte maturity (r = −0.212, *p* = 0.048). Furthermore, there was a strong negative connection between CART expression and thyroid hormone T3 (r = −0.319, *p* = 0.048). Among FSHR polymorphisms, the SER/SER genotype was related to greater CART levels (mean 4.198 ± 2.257) than the SER/ASN and ASN/ASN genotypes (*p* = 0.031). Conclusions: These data indicate that CART expression and FSHR polymorphisms may influence ovarian response and oocyte quality in IVF patients, possibly acting as biomarkers for evaluating ovarian outcomes in various ovulation induction procedures.

## 1. Introduction

Research has shown that the environment of the follicular fluid (FF) is strongly associated with oocyte maturity [1]. Therefore, the hormone profile within the FF might indicate oocyte maturity, although this relationship is not yet fully established. Since hormones mediate various stages of folliculogenesis, their levels could potentially indicate oocyte viability, fertilization potential, and embryo development.

Leptin, a hormone mostly produced by adipose tissue, regulates energy homeostasis by interacting with particular receptors in the hypothalamus [2]. Leptin dysregulation in the setting of obesity might interfere with gonadotropin-releasing hormone (GnRH) release, which can impact the generation of reproductive hormones and possibly result in infertility [3]. By promoting the expression of the CART neuropeptide, leptin also has a notable effect on the brain, indicating a noteworthy connection between these two regulatory elements [4]. Furthermore, leptin stimulation raises CART expression, which suppresses steroidogenesis and GC aromatase expression [5]. The expression of the CART neuropeptide in the brain is activated and follicle development is directly influenced by leptin receptors in the ovaries. Moreover, the growth of ovarian follicles is directly influenced by leptin receptors found in the ovaries [6]. Obese patients’ ovarian granulosa cells (GCs) have elevated CART levels, with leptin enhancing CART expression, which suppresses GC aromatase production and steroidogenesis.

FF is essential for oocyte maturation as it mainly includes steroid hormones, cytokines, antioxidants, and growth factors [7,8], playing a major role in the communication between oocytes and their surrounding cells. Additionally, FF provides amino acids, lipids, and nucleotides for oocyte maturation influencing fertilization and early embryo development. Many studies have proposed that the amount and composition of metabolites in FFs collected from patients with various infertility disorders, such as PCOS [9,10], and diminished ovarian reserve (DOR) [7], are different compared to healthy controls [11]. Consequently, FF provides a broad picture of the biological state of the oocytes [12].

To address fertility concerns, reproductive medicine has devised a variety of ovulation induction procedures. These protocols investigate several forms of gonadotropins, which are either taken from pure human urine or manufactured using recombinant techniques. Urine possibilities include Human Menopausal Gonadotropin (HMG) [13], urine FSH (uFSH) [14], and urinary hCG (uhCG) [15], while recombinant alternatives include rFSH [16,17], rLH [18], and rhCG [19]. Despite extensive study, deciding whether uFSH or rFSH is more successful remains difficult, since studies have found no significant differences in oocyte retrieval or pregnancy rates between the two.

These gonadotropins are used to produce controlled ovarian stimulation (COS) in women undergoing Assisted Reproductive Technology (ART). However, no one agrees on the optimal protocol. Some studies propose combining LH-related therapies with FSH in COS, and LH, hCG, HMG, or their mixtures have been utilized with or without FSH in COS [20,21,22].

Researchers have also investigated the relationship between polymorphisms at position 680 of the FSHR gene and results in Assisted Reproductive Technology. A prominent polymorphism is the substitution of guanine for adenine (c.2039 G>A) in exon 10 (rs6166), which results in an amino acid change from serine to asparagine at position 680 (Asn680Ser) [23,24]. Studies have shown significant variations in responses to Controlled Ovarian Stimulation (COS) during IVF or intracytoplasmic sperm injection (ICSI) protocols based on the Ser680Asn genotypes (G/G—Ser/Ser, G/A—Ser/Asn, A/A—Asn/Asn), either as standalone factors or in combination with polygenic analyses of polymorphisms in the Estrogen Receptor 1 (ESR1) and Estrogen Receptor 2 (ESR2) genes [25,26].

A recent meta-analysis looked at the effects of various FSHR Ser680Asn genotypes on COS results in IVF/ICSI patients. The study found that women with the Asn/Asn genotype had greater estradiol (E2) levels on the day of hCG injection but generated fewer embryos for transfer than women with the Ser/Ser genotype. Similarly, women with the Ser/Asn genotype exhibited higher E2 levels on that day of hCG administration [27].

This study investigates, for the first time, the association between CART and leptin with hormone profiles in follicular fluid derived from four distinct ovulation induction protocols, including progesterone, testosterone, androstenedione, estradiol, FSH, and hCG. In addition, the study aims to investigate the relationship between FSHR Ser680Asn genotypes (Ser/Ser, Ser/Asn, and Asn/Asn), CART, and leptin in the context of IVF outcomes.

## 2. Materials and Methods

The study was undertaken at the Diagnostic and Therapeutic Fertility Institute S.A. in Athens, Greece, from April of 2022 to June of 2024. In this trial, 94 women between the ages of 24 and 45 received GnRH antagonist-based controlled ovarian stimulation. Patient recruitment was accomplished using a computer-generated randomization table. Four treatment groups were assigned to the participants: rFSH (*n* = 29) (Gonal-F Merck), HMG (*n* = 21) (Menopour Ferring), HMG/hCG (*n* = 23), and rFSH/hCG (*n* = 21). The hCG (Pregnyl MSD) was given to the participants at a low dose of 100 IU/day. The patient’s age, AMH, FSH, LH, and antral follicle count (AFC) were taken into consideration when selecting the treatment plan. Given that older women’s pregnancy outcomes are improved by LH or hCG activity, women under 35 received rFSH, and those over 35 received either HMG, rFSH + hCG, or HMG + hCG [28].

Eligibility criteria included the absence of uterine or ovarian abnormalities, a normal hormonal profile according to WHO recommendations, a 25–30-day menstrual cycle, and undamaged ovaries. Male factor, tubal factor, and infertility without apparent cause were among the criteria for fertility treatment. Before the trial, for at least three months, none of the subjects had received hormone treatment or ovarian stimulation. The obtained data included anthropometric parameters (age and BMI), as well as early follicular phase values of FSH, LH, PRL, AMH, TSH, T3, T4, TPO, TG, A, and DHEA-S from the previous six months. The Diagnostic and Therapeutic Fertility Institute S.A. review board approved the study protocol (approval number 11/2020, dated 20 December 2020). Every participant gave their informed consent for the use of their medical records.

### 2.1. Ovarian Stimulation Protocol

In accordance with our institution’s standard operating protocols, patients in this trial underwent controlled ovarian stimulation after a GnRH antagonist protocol. They were given daily doses of the GnRH antagonist Orgalutran (MDS, Kalamaki, Greece) starting on the fifth day of the menstrual cycle until the oocytes’ ultimate maturation was triggered by rhCG (Ovitrell, Merck, Marousi, Greece). On the second day of the cycle, gonadotropin therapy was started at a 200 IU dosage, and daily changes were made based on the ovarian response. In addition, intramuscular hCG (Pregnyl, MSD Hellas) was injected once daily at a dose of 100 IU starting on day 2 of the cycle and continued until the last maturation trigger. From the seventh day of the cycle, on the fifth day of gonadotropin treatment, until the final maturation trigger with 250 μg of rhCG (Ovitrell, Merck Hellas), the levels of serum estradiol (E2) were tracked every day. On the eighth day of the cycle, or the sixth day of stimulation, follicular monitoring was initiated. Up to oocyte retrieval, daily ultrasound scans were carried out.

### 2.2. Follicular Fluid Sample

Transvaginal ultrasound-guided puncture was performed for follicular aspiration and oocyte retrieval 36 h following the rhCG trigger. Each follicle was manually aspirated with a 20 mL syringe and a single-lumen needle (Cook Medical, Bloomington, IN, USA). Follicular fluid (FF) from follicles with a minimum diameter of 12 mm was collected and centrifuged, and the supernatants were separated and kept at −20 °C for future study. Following this, IVF and ICSI operations were carried out. Embryo quality was evaluated using specific criteria, including blastomere count, degree of fragmentation, blastomere consistency, and multinucleation.

The embryo transfer took place on the fifth day following the oocyte retrieval. 200 mg of micronized progesterone was delivered intravaginally three times daily commencing the day following egg harvest to support the luteal phase. Serum beta-hCG levels were tested 14 days after transfer. At six weeks of gestation, an ultrasound revealed a gestational sac, confirming a clinical pregnancy. One of the clinic’s two fertility experts conducted all embryo transfers, oocyte retrievals, and ultrasound exams. One of the institute’s two senior embryologists oversaw the fertilization, oocyte grading, early embryo development, and embryo grading.

### 2.3. Follicular Fluid Hormonal Measurement

The Laboratory Genes Lab in Athens, Greece, conducted all hormone studies on follicular fluids (FFs), which included progesterone (Prg), testosterone (T), androstenedione (A), hCG, FSH, and estradiol (E2). Progesterone and estradiol needed to be diluted at a ratio of 1:1000. The androstenedione was analyzed using the RIA method, and the other hormones were analyzed using the COBAS 6000 analyzer (COBAS 6000, Roche Diagnostics, Ludwigsburg, Germany). Each assay had the following detection limits: hCG 0.1 IU/L; FSH 0.1 IU/L; LH 0.1 IU/L; E2 0.02 nmol/L; progesterone 0.1 nmol/L; testosterone 0.087 nmol/L; and androstenedione 0.1 nmol/L.

### 2.4. Genotyping

Peripheral blood was collected from study participants to perform FSHR Ser680Asn genotyping analysis. The samples were stored at −20 °C. DNA isolation was conducted using the PureLink Genomic DNA kit (Invitrogen, Carlsbad, CA, USA), following the manufacturer’s instructions. Real-time polymerase chain reaction (RT-PCR) was applied for the detection of Ser680Asn polymorphism, using the LightCycler 480II (RocheGmBH, Manheim, Germany). The sequences of the FSHR-specific primers and probes used were as follows: FSHR S AGTGTGGCTGCTATGAAATGC, FSHR A GGCTAAATGACTTAGAGGGACAAGTA, SP.

### 2.5. Detection of Leptin and CART Gene Expression

Follicular fluid samples were collected from all the participants, and the samples were kept at −80 °C until the RNA was extracted. Total RNA was isolated from blood samples using the Monarch Total RNA miniprep kit supplied by New England Biolabs (Ipswich, MA, USA). One μg of the extracted RNA was used to synthesize complementary DNA (cDNA) using the LunaScript RT SuperMix, (New England Biolabs). We measured the expression of the CART and leptin genes using real-time polymerase chain reaction (RT-PCR) with 5 μL of cDNA. The LightCycler480 II device from Roche Life Sciences was used for all RT-PCR experiments, and the Luna Universal qPCR Master Mix from New England Biolabs was used at a final concentration of 1×. The CART gene primer sequences were 5′GCTGAAGAAGCTTTGAAGAAGC3′ (Eurofins Genomics GmBH, Ebersberg, Germany) for the forward primer and 3′GCACTTCAGGAGGAAGGAATTGC5′ (Eurofins Genomics GmBH) for the reverse primer. The primers for the leptin gene were 5′GAACCCTGTGATTCTT3′ for the forward primer (Eurofins Genomics GmBH) and 5′CCAGGTCGTTATTTGG3′ (Eurofins Genomics GmBH) for the reverse primer. An initial denaturation phase at 94 °C for one minute was followed by 40 cycles of denaturation at 95 °C for fifteen seconds and annealing/extension at 60 °C for thirty seconds in the PCR reaction. A melting curve detection was then carried out to confirm the specificity of the reaction. The housekeeping gene G6PD was used as a reference gene. Every assay was performed twice, and a negative control was added. The 2^−∆∆CT^ technique was applied to detect the relative mRNA expression levels of CART and leptin genes.

### 2.6. Sample Size Determination

A statistical power analysis was conducted to determine the appropriate sample size for our research, ensuring that the study had sufficient statistical power to detect significant differences in outcomes. To determine the impact size for the power analysis, we carefully reviewed the recent literature and cited relevant research. We calculated a modest effect size (Cohen’s d = 0.5) for the primary outcome measures, CART and leptin gene expression, based on this literature evaluation and expert opinion. This impact size was selected as a conservative approximation that could detect significant changes in gene expression. The statistical power level of 80% (1 − β = 0.80) was chosen to optimize the likelihood of identifying actual effects, should they exist. The significance level (α) was chosen to be 0.05, representing a 5% possibility of a type I mistake. We performed the power study using statistical software (G*Power, version 3.1.9.7) with these settings. According to the research, in order to obtain the appropriate statistical power, a total sample size of 89 participants was required. To guarantee the study’s viability in a clinical setting, pragmatic considerations including patient availability and resources were taken into account.

### 2.7. Statistical Analysis

Several statistical approaches were used to determine the associations between dCp CART expression and different clinical, hormonal, and genetic indicators in individuals undergoing in vitro fertilization (IVF). A thorough explanation of the tests used is provided below:

Pearson’s correlation coefficient was used to establish the linear relationship between dCp CART levels and a variety of clinical and hormonal indicators throughout the cohort. This test determines the degree and direction of the linear relationship between two continuous variables and returns a correlation coefficient (r) ranging from −1 to +1. A score closer to +1 suggests a strong positive association, whereas a value closer to −1 indicates a strong negative relationship. A *p*-value less than 0.05 was deemed statistically significant for this analysis.

Kendall’s Tau-B Correlation: given the ordinal nature of some data, including the possibility of ties between hormone levels and stimulation protocols, Kendall’s tau-b correlation was used to determine the strength of association between CART gene expression and hormonal markers for each protocol group (HMG, HMG/hCG, recombinant FSH, recombinant FSH /hCG). This non-parametric test assesses the correlation between two ranking variables and is especially reliable when the assumption of normality is broken if the data contains ties. This strategy enabled a more accurate analysis within each stimulation regimen. A *p*-value of less than 0.05 was considered statistically significant, similar to Pearson’s correlation.

To evaluate dCp CART levels between different IVF stimulation regimens and FSH receptor polymorphisms, an independent samples t-test was used. This parametric test determines if there is a statistically significant difference in the means of dCp CART levels among two independent groups. Separate analyses were performed within each protocol group, allowing for comparisons of the HMG, HMG/hCG, rFSH, and rFSH/hCG regimens, as well as FSH receptor polymorphisms (SER/SER, SER/ASN, ASN/ASN). For comparisons between more than two groups or when polymorphisms were merged into subgroups, the same independent samples t-test was used to analyze variations in CART expression. All comparisons were judged statistically significant with a *p*-value of <0.05.

All statistical analyses were carried out using IBM SPSS Statistics (version 20). This program was chosen because of its strong ability to execute both parametric and non-parametric tests, assuring data accuracy and result interpretation across a wide range of clinical, hormonal, and genetic datasets.

## 3. Results

Of the 94 women who initially registered in the research, 93 continued as planned, while one withdrew for personal reasons. The power analysis indicated that a minimum of 89 individuals was necessary to maintain appropriate statistical power.

Table 1 presents the demographic characteristics of the research participants. In terms of age, the bulk of the women in the sample (74.2%) were between the ages of 31 and 40, with a lower proportion being younger than 30 (5.4%) or older than 40 (19.4%). In terms of body weight, the majority of participants (65.2%) were between 55 and 70 kg, with 15.2% weighing less than 55 kg, 12.0% between 70 and 85 kg, and 7.6% weighing more than 85 kg.

According to Table 1, in terms of years of infertility, 71.7% of women reported being infertile for 0–5 years, 20.7% for 6–10 years, and 7.6% for more than 10 years. The number of prior IVF tries indicated that the great majority (90.2%) had had 0–2 previous attempts, 7.6% had 3–4 attempts, and 2.2% had more than 4 attempts. Participants reasons for IVF varied, with 62.0% of the sample getting IVF for male factor infertility, 32.6% for unexplained infertility, 4.3% for tubal causes, and 14.74% for elevated FSH levels.

Hormonal levels were also determined, with the mean FSH level at 7.45 mIU/mL (SD = 2.49), the mean LH level at 6.55 mIU/mL (SD = 2.94), the mean prolactin level at 16.36 ng/mL (SD = 6.74), and the mean AMH level at 3.24 ng/mL (SD = 1.94). The average number of stimulation days was 9.68 (standard deviation = 1.5). In terms of follicles larger than 12 mm, 60.9% of individuals had 6–10 follicles, 35.9% had more than 10 follicles, and just 3.3% had less than 5. The mean number of eggs collected was roughly 9 (SD = 2.61), with an average of 8 mature eggs retrieved. The average number of embryos created was 7 (SD = 2.05), with the majority of embryos classed as “good” (75.0%), 20.7% as “excellent”, and 4.3% as “poor”. The average number of embryos transferred was two (standard deviation = 6.29). On the day of egg collection, endometrial thickness ranged from 8.1 to 10 mm in 68.5% of women, <8 mm in 17.4%, and 10.1–12 mm in 14.1%. Finally, the pregnancy rate showed that 24.7%, 23/93 of women became pregnant.

Table 2 indicates the frequency of the regimes. The protocol was assessed as a qualitative variable, with four categories depending on the pharmacological regimen delivered to each woman: (a) HMG/hCG, (b) rFSH-hCG, (c) rFSH, and (d) HMG. More specifically, as indicated in Table 1, 25.8% of the women in the sample received the HMG/hCG treatment, 21.5% received the rFSH-hCG protocol, 30.1% received the rFSH protocol, and 22.6% received the HMG protocol.

Table 3 shows the correlation between BMI and CART expression. The Pearson correlation coefficient between BMI and dCp CART values is −0.011, with a *p*-value of 0.914. This indicates that there is no significant correlation between BMI and the expression of the cocaine-amphetamine-regulated transcript (CART) gene.

Table 4 shows the statistical analysis performed using both parametric and non-parametric tests to evaluate the relationships between body mass index (BMI) and various parameters. Parametric tests, including Pearson correlation and *t*-tests, were utilized to assess linear relationships and group differences among normally distributed data. In contrast, non-parametric tests, such as the Mann–Whitney U test, were applied for comparisons between groups when the assumptions of normality were not met. *p*-values of less than 0.05 were considered statistically significant.

The statistical analysis shown in Table 4 compares BMI, ovulation induction procedures, pregnancy outcomes, FSHR polymorphisms, and a number of hormonal and reproductive factors between groups. There were no statistically significant variations in BMI amongst the four ovulation induction procedures (HMG, HMG/hCG, rFSH, and rFSH/hCG), with *p*-values ranging from 0.244 to 0.909, showing that BMI distributions were similar across treatments. In terms of pregnancy outcomes, there was no significant difference in BMI between women who became pregnant and those who did not (*p* = 0.625), indicating that BMI was not a decisive factor for pregnancy success in this cohort.

The mean BMI changed across the ASN/ASN, SER/ASN, and SER/SER genotypes for FSHR polymorphisms, but the differences were not statistically significant (*p*-values ranged from 0.218 to 0.959). Furthermore, no significant difference was identified between the combined SER/SER + SER/ASN group and the ASN/ASN group (*p* = 0.588 and 0.821, respectively). There were no significant connections found between hormonal variables such as FSH, LH, AMH, testosterone, and androstenedione, with androstenedione having the lowest *p*-value (*p* = 0.096). Similarly, the study of follicular response, as measured by the number of follicles and mature MII oocytes, revealed no statistically significant changes (*p*-values of 0.75 and 0.956, respectively). Pearson’s correlation coefficient was used to determine the linear association between dCp CART and a variety of clinical and hormonal markers.

In this study, the relationship between dCp CART and several clinical and hormonal markers in IVF patients was examined. According to Table 5, a strong negative association was found between dCp CART and T3 levels (r = −0.319, *p* = 0.048), indicating a possible relationship between thyroid function and the dCp CART index.

Furthermore, there was a significant negative relationship between dCp CART and the number of follicles bigger than 12 mm (r = −0.251, *p* = 0.018), total oocytes recovered (r = −0.247, *p* = 0.019), and oocyte maturity at the MII stage (r = −0.212, *p* = 0.048). These findings suggest that increased dCp CART levels may potentially affect follicular growth and oocyte quality under controlled ovarian stimulation.

Although the association between dCp CART and the number of embryos was not statistically significant (r = −0.206, *p* = 0.056), the pattern implies a potential link with the number of embryos. Other variables, such as the number of gonadotropins and stimulation days, did not have significant relationships with dCp CART, suggesting that the observed effects are more likely due to patient-specific physiological factors than changes in stimulation regimens.

Table 6: The Pearson correlation coefficient was utilized to analyze the linear association between CART gene expression and hormone levels throughout the entire cohort. Because of the ordinal character of the data or the occurrence of ties, Kendall’s tau-b correlation was used for each protocol (HMG, HMG/hCG, rFSH, rFSHL/hCG) to assess the strength of the connection between CART gene expression and hormone levels. *p*-values less than 0.05 were deemed statistically significant.

Table 6 shows the correlation coefficients and *p*-values for the relationship between the expression of the CART (Cocaine- and Amphetamine-Regulated Transcript) gene and various hormone levels across four different IVF treatment protocols: HMG, HMG/hCG, rFSH, and rFSH/hCG, as well as for the entire patient population.

There were no statistically significant relationships found between CART expression and measured hormone levels (progesterone, testosterone, androstenedione, hCG, FSH, and E2). The *p*-values were all higher than 0.05, while the correlation coefficients were comparatively low, indicating weak relationships. The greatest association in the group overall was found between CART and testosterone, with a coefficient of 0.131 and a *p*-value of 0.239.

In the separate treatment protocols, Protocol 1 (HMG) showed minor relationships between CART expression and hormones, none of which were significant. The strongest connection in this protocol was seen with FSH, with a coefficient of 0.257 and a *p*-value of 0.139. Similarly, Protocol 2 (HMG/hCG) showed no significant relationships. The highest correlation in this group was with FSH, which had a coefficient of 0.222, although the *p*-value of 0.173 suggests that this association was not statistically significant.

Protocol 3 (rFSH) followed a similar pattern, with no statistically significant relationships detected. The highest negative association in this protocol was between CART and FSH, with a coefficient of −0.269 and a *p*-value of 0.055, which neared statistical significance but fell short of the threshold. In contrast, Protocol 4 (rFSH/hCG) found a statistically significant positive correlation between CART expression and FSH, with a coefficient of 0.448 and a *p*-value of 0.008, indicating a relationship. Although the other hormones had no significant associations in this experiment, testosterone showed a trend toward significance, with a correlation coefficient of 0.305 and a *p*-value of 0.069.

In Table 7, an independent samples *t*-test was utilized to compare the means of two groups within each stimulation regimen when analyzing dCp CART levels.

Table 7 indicates that the dCp CART levels in IVF patients were compared using various stimulation protocols. When comparing treatments using HMG/hCG with rFSH/hCG, the dCp CART values differed significantly (*p* = 0.029). The HMG/hCG group had a greater mean dCp CART (4.106 ± 2.100) than the rFSH/hCG) group (3.165 ± 1.898), indicating that the type of gonadotropin utilized with hCG affects dCp CART levels.

Further research was carried out by comparing the dCp CART results for each technique separately. Protocol 2 (HMG/hCG) had a substantially higher mean dCp CART value (4.424 ± 1.922) compared to the other protocols (3.342 ± 2.019) (*p*-value = 0.030). There were no statistically significant differences between Protocol 1 (HMG) (*p* = 0.715), Protocol 3 rFSH (*p* = 0.443), and Protocol 4 (rFSH/hCG) (*p* = 0.080). These findings indicate that the combination of HMG and HCG has a larger influence on dCp CART levels than other gonadotropin regimens, which may have implications for ovarian stimulation techniques in IVF procedures.

Table 8: To compare the dCp CART values between the FSH receptor polymorphism groups, an independent samples *t*-test was used. This test was used to determine whether the means of the two groups were statistically distinct from one another. For comparisons involving more than two groups (i.e., combined polymorphisms), other groupings were evaluated using the same procedure. A *p*-value of <0.05 was judged statistically significant.

Table 8 shows the average dCp CART values for various FSH receptor polymorphisms. The SER/SER group had a higher mean dCp CART value (4.198 ± 2.257) than the SER/ASN or ASN/ASN groups, but the difference was not statistically significant (*p* = 0.098). For FSHR Variant 1, when SER/SER and SER/ASN were combined and compared to ASN/ASN, there was no significant change in mean dCp CART values (*p* = 0.456). Similarly, in FSHR variation 2, the combination of SER/SER vs. SER/ASN + ASN/ASN indicated a statistically significant difference (*p* = 0.031), indicating that the SER/SER variation is related with greater dCp CART levels than the combined group. However, there was no significant difference between SER/SER + ASN/ASN vs. SER/ASN (FSHR Variant 2) (*p* = 0.113). These data indicate that the SER/SER variation may contribute to increased dCp CART levels, although the magnitude of the difference varies depending on how the groups are merged for analysis.

## 4. Discussion

This is the first study in which, the expression of leptin and CART was examined in the FF of ovarian follicles obtained from patients undergoing four distinct multiple ovulation induction protocols: HMG, HMG-hCG, rFSH, and rFSH-hCG.

Furthermore, the study examined the relationship between CART dCp levels and the quantity and quality of oocytes and embryos. The link between CART expression hormonal profile, and polymorphisms in the Follicle-Stimulating Hormone Receptor (FSHR) gene was also investigated. Additionally, this study also analyzed several clinical characteristics linked with CART expression in follicular fluid, including ovulation induction regime, body mass index (BMI), hormonal profiles, and FSHR polymorphisms, to provide insights into their possible involvement in reproductive outcomes.

The study yielded a correlation value of −0.011 between BMI and dCp CART, with a *p*-value of 0.914, showing that BMI has no meaningful association with the expression of the CART gene. Our findings showed that leptin levels in the follicular fluid of women undergoing gonadotropin ovulation induction were very low. These findings are comparable with those of Xiaoting Ma et al., who found a favorable connection between CART levels in follicular fluid and BMI [5].

A significant negative correlation was found between dCp CART and T3 levels indicating a possible relationship between thyroid function and dCp CART expression. Furthermore, a significant negative correlation was found between dCp CART and several ovarian response markers, including the number of follicles larger than 12 mm, total oocytes retrieved, and mature oocytes at the metaphase II (MII) stage. These data imply that lower dCp CART levels may be linked with a better ovarian response, potentially impacting follicular development, oocyte retrieval, and oocyte maturity. The correlation between dCp CART and the number of embryos was not statistically significant, the findings indicate that CART does not have a utility in the post-fertilization stage and early embryo development. This finding aligns with a study by Ma et al. that emphasized the relevance of CART in reproductive processes in human IVF [5].

We also examined the correlation between the expression of the CART gene and various hormone levels in follicular fluid. No statistically significant relationships were discovered between CART expression and measured hormone levels (progesterone, testosterone, androstenedione, hCG, FSH, and estradiol (E2). Similarly, in the HMG and HMG/hCG regimens, there were no significant associations between CART expression and hormonal levels. However, the rFSH gonadotropins preparation revealed a link between CART and FSH, level with a correlation coefficient of −0.269 and a *p*-value of 0.055. Notably, the rFSH/hCG regime revealed a statistically significant positive correlation between CART expression and FSH, with a correlation coefficient of 0.448 and a *p*-value of 0.008, indicating a substantial relationship.

The significant number and functional variety of genes whose expression differs across rFSH and HMG regimens might explain the observed association in the rFSH regimen. In a recent study, 85 genes were shown to be differently expressed in granulosa cells (GC) from women treated with either rFSH or HMG. Differences also exist in the FSH isoform profiles and possibly in the specific activity of FSH, which could influence the variations in gene expression [28,29]. These gene expressions and the isoform changes may have an impact on the ovarian response, follicular growth, and hormone levels, which might explain the strong association between CART expression and FSH seen in the rFSH and rFSH/hCG protocols [30].

We measured dCp CART levels in IVF patients under various stimulation protocols. An independent samples *t*-test was conducted to compare the mean dCp CART levels between two groups within each protocol. Important differences in dCp CART levels were found in the HMG/hCG and rFSH/hCG groups (*p* = 0.029 and *p* = 0.080, respectively) as compared with other protocols (Table 7). The HMG/hCG group had a higher mean dCp CART value (4.106 ± 2.100) compared to the rFSH/hCG group (3.165 ± 1.898), suggesting that the type of gonadotropin used alongside hCG influences dCp CART levels. These results suggest that the combination of HMG and hCG has a greater impact on dCp CART levels compared to other gonadotropin regimens, which could have implications for ovarian stimulation strategies in IVF procedures.

Multiple studies have demonstrated the advantages of hCG-based ovulation induction protocols [31]. The structural differences in carbohydrate content enhance hCG’s affinity for binding receptors. Additionally, hCG has a longer plasma half-life of approximately 24–33 h [32] compared to 10–12 h for LH, leading to more sustained and effective ovarian stimulation [33]. This extended half-life, along with hCG’s greater potency (about six to eight times higher than LH), results in more prolonged and effective occupancy of LH/hCG receptors.

Increasing the amount of hCG supplementation significantly increased the intrafollicular concentration of both estradiol and androgens, resulting in a more androgenic environment. Large follicles with good-quality embryos have considerably more estrogenic FFs than small follicles with poor-quality embryos [34]. The CART gene may interact with the presence of hCG to regulate reproductive hormone pathways, ovarian function, and neuroendocrine feedback mechanisms.

An interesting observation in the study was the association of FSHR receptor polymorphisms with dCp CART values. The Ser/Ser phenotype has a higher mean dCp CART value (4.198 ± 2.257) than the SER/ASN or ASN/ASN groups. The combination of SER/SER vs. SER/ASN + ASN/ASN indicated a statistically significant difference (*p* = 0.031), indicating that the SER/SER variation is related to greater dCp CART levels than the combined group. The observation that the Ser/Ser genotype presents a higher value of dCP CART suggests some correlation of CART with this phenotype. The FSHR receptor plays a crucial role in folliculogenesis and ovarian response. Polymorphisms, particularly Ser/Ser, have been linked to varying ovarian responses to FSH stimulation. It has been shown that SNPs of the FSHR gene are associated with an altered FF milieu. resulting in significantly increased androgen levels [35]. Zhou et al. studied alternatively spliced FSHR-2 and FSHR-3 and showed that both isoforms were expressed at a low level, in the follicular fluid but they did not establish a possible association between the presence of SNPs and the follicular fluid [36].

A meta-analysis supports that the Ser/Ser genotype is associated with higher numbers of embryos produced and transferred, suggesting that this variant may affect ovarian sensitivity to FSH, leading to enhanced follicular development [27]. The correlation between Ser/Ser genotype and higher dCp CART levels might suggest that CART could be influencing FSH signaling or ovarian function more effectively in this genetic background. CART’s role in modulating reproductive hormone sensitivity, or energy regulation, may be more pronounced in individuals with the Ser/Ser FSHR variant, enhancing fertility-related outcomes. The Ser/Ser FSHR polymorphism might influence reproductive success via its association with higher CART expression or activity, which in turn could positively modulate ovarian response and fertility outcomes. Further studies would be needed to clarify the exact biological interaction between CART and FSHR in this context.

Leptin stimulates oocyte maturation via the MAPK (Mitogen-Activated Protein Kinase) signaling pathway, which is essential for cellular functions such as growth, differentiation, and survival [37]. Mantzoros et al. discovered that lower levels of leptin in both blood and follicular fluid were related to increased pregnancy rates, indicating that excessive leptin may represent metabolic abnormalities that impair oocyte quality and embryo implantation [38]. Similarly, Tsai et al. show that high leptin levels were associated with lower pregnancy rates and poor steroidogenesis in granulosa cells, which are required for oocyte maturation and embryo development. Their findings showed that leptin can inhibit the synthesis of steroid hormones such as estrogen and progesterone, potentially disrupting the ovarian environment required for successful conception and implantation [39]. Zachow et al. have shown that leptin has a negative influence on ovarian steroidogenesis, specifically by inhibiting estradiol (E2) production [40]. Agarwal et al., in their research, have shown that elevated leptin levels inhibit FSH-mediated activity. This suppression alters important signaling pathways, including the insulin-like growth factor 1 (IGF-1) axis, leading to impaired estradiol production and compromised folliculogenesis and ovulation processes [41]. Furthermore, IGF-1 competes for insulin receptors, limiting downstream activation of JAK/STAT signaling, which leptin generally uses to achieve its effects. This further reduces leptin activity in the ovarian microenvironment [42].

The interaction of CART and leptin in obesity appears to be synergistic in the setting of reproductive dysfunction [5]. CART expression in granulosa cells and follicular fluid has been linked to BMI, with obese women showing greater CART levels [43]. This shows that CART may contribute to obesity-induced changes in ovarian function, possibly through leptin interaction and gonadotropin control. Elevated CART levels in the ovarian microenvironment may aggravate obese women’s already poor leptin signaling, compromising normal ovarian physiology. This disturbance can have an impact on important reproductive processes including follicular recruitment, oocyte maturation, and hormone synthesis, all of which are required for proper fertilization and embryo development.

At the molecular level, CART interacts with a number of pathways that affect ovarian function [44]. One such mechanism is the MAPK signaling cascade, which is known to control several aspects of cell growth and development. In the context of ovarian physiology, CART has been demonstrated to activate MAPK, which can then reduce aromatase mRNA expression in granulosa cells [45]. Aromatase is a critical enzyme that converts androgens into estradiol, a hormone required for follicular growth and oocyte maturity. Reduced aromatase expression causes decreased estradiol levels, which hinders normal follicular development and results in fewer ovulated oocytes [36]. This reduction in estradiol production, along with the deleterious consequences of leptin resistance and decreased gonadotropin signaling, may cause subfertility or infertility, particularly in women with obesity.

The suggested hypothesis of CART-mediated obesity-related infertility proposes that CART acts as a mediator of ovarian metabolic dysfunction, impacting leptin’s capacity to control gonadotropin signaling and estradiol synthesis. Overexpression of CART in obese women’s ovarian tissue adds to persistent low-grade inflammation and metabolic stress, which, when paired with leptin resistance, further impairs ovarian function. Elevated CART, decreased aromatase activity, decreased estradiol production, and disrupted leptin signaling all contribute to suboptimal reproductive outcomes in women undergoing assisted reproductive technologies (ART), such as IVF. Understanding these molecular pathways shows the relevance of the CART-leptin axis in obesity-related infertility and gives possible treatment targets to improve reproductive performance outcomes in obese women.

## 5. Conclusions

These data imply that increased dCp CART levels, which indicate a better affinity for the CART receptor, may be associated with enhanced ovarian response, potentially impacting follicular development, oocyte retrieval, and maturation of oocytes. In protocols utilizing recombinant FSH and rFSH/hCG, a relationship between CART and FSH was revealed. Furthermore, CART expression was considerably greater in all hCG-containing protocols, with the HMG/hCG protocol showing the most marked rise, indicating that CART may play a role in ovarian stimulation.

The finding that the Ser/Ser genotype is associated with greater dCp CART levels supports a link between CART expression and this genetic variation. Elevated dCp CART levels when carrying the Ser/Ser genotype may indicate a unique connection between CART signaling pathways and the Ser/Ser variation of the FSHR receptor. Given that individuals with the Ser/Ser genotype have been demonstrated to have superior reproductive results, such as a larger number of embryos transferred, the increased dCp CART levels might be due to improved CART gene activity in response to or in combination with the Ser/Ser FSHR variation. This interaction may contribute to the better ovarian responses observed in those with the Ser/Ser genotype. Additionally, the evaluation of follicular fluids requires further research. It seems important to study individual follicular fluids making a more personalized treatment possible by individualizing the culture media and consequently improving embryo development.

## Figures and Tables

**Table 1:** Demographic Characteristics of the Study Sample.

DemographicVariables	Categories	Frequency	Percentage (%)
**Age**	<30 years	5	5.4
	31–40 years	69	74.2
	>40 years	18	19.4
**Weight (kg)**	<55 kg	14	15.2
	55–70 kg	60	65.2
	70–85 kg	11	12.0
	>85 kg	7	7.6
**Years of Infertility**	0–5 years	66	71.7
	6–10 years	19	20.7
	>10 years	7	7.6
**Number of PreviousAttempts**	0–2	83	90.2
	3–4	7	7.6
	>4	2	2.2
**Reason for IVF**	Tubal Factor	4	4.3
	Elevate FSH	1	14.74
	Male Factor	57	62.0
	Unexplained Infertility	30	32.6
**Number of Follicles (>12 mm)**	<5	3	3.3
	6–10	56	60.9
	>10	33	35.9
**Embryo Quality**	Excellent	19	20.7
	Good	69	75.0
	Poor	4	4.3
**EndometrialThickness (mm)**	<8 mm	16	17.4
	8.1–10 mm	63	68.5
	10.1–12 mm	13	14.1
**PregnancyOutcome**	No	93	75.3
	Yes	23	24.7

**Table 2:** Frequency table for the stimulation protocol.

Stimulation Protocol	Frequency	Percentage (%)
HMG/hCG	24	25.8
rFSH/hCG	20	21.5
rFSH	28	30.1
HMG	21	22.6
Total	94	100

**Table 3:** BMI association with dCp CART.

Correlation Between	Statistic	Value
BMI and dCpCART	PearsonCorrelation	−0.011
BMI and dCpCART	*p*-value	0.914

**Table 4:** The statistical analysis of BMI and various parameters.

Variable/Protocol	Mean	StdDev	*p*-Value
HMG (1)	23.10	3.58	0.244
HMG/HCG (2)	24.26	5.25	0.909
r-FSH (3)	23.66	4.41	0.575
r-FSH/HCG (4)	25.66	5.93	0.274
NoPregnancy (0)	24.27	5.04	0.625
Pregnancy (1)	23.68	4.28	0.625
ASN/ASN	24.91	4.86	0.959
SER/ASN	23.39	4.19	0.290
SER/SER	24.81	5.59	0.218
Group A (ASN/ASN)	24.91	4.86	0.588
Group B (SER/SER + SER/ASN)	23.99	4.85	0.821
FSH mIU/mL	7.816	3.238	0.837
LH mIU/mL	6.562	3.077	0.928
AMH ng/mL	3.974	6.538	0.929
Testosterone ng/dL	430.208	389.165	0.309
Androstenedione ng/mL	4.044	1.584	0.096
Follicles	9.859	3.052	0.755
Mature MII	8.047	2.895	0.956

**Table 5:** Association between dCp CART and Various Clinical Parameters in IVF Patients.

Variable	Pearson Correlation	*p*-Value
Age	0.066	0.541
Sterility	−0.116	0.278
Trials	−0.204	0.055
Weight kg	−0.127	0.235
Height cm	0.069	0.520
FSH mIU/mL	−0.058	0.590
LH mIU/mL	−0.116	0.280
PRL mIU/mL	0.019	0.863
AMH ng/mL	−0.019	0.864
TSH µIU/mL	−0.022	0.837
T3 ng/dL	−0.319	0.048
T4 µg/dL	−0.203	0.216
FT3 pg/mL	−0.146	0.417
FT4 ng/dL	0.060	0.666
a-TPO IU/mL	0.098	0.511
a-TG IU/mL	−0.013	0.934
Total Drug Units	0.013	0.907
Stimulation Days	−0.013	0.902
E2 on Day of HCG	−0.053	0.621
Nofollicles > 12 mm	−0.251	0.018
Number of oocytes	−0.247	0.019
Maturity MII	−0.212	0.048
Embryo	−0.206	0.056

**Table 6:** Correlation coefficients and *p*-values between CART gene expression and hormone levels in different IVF treatment protocols.

Protocol	Hormone	Correlation Coefficient	*p*-Value
Overall	Progesterone ng/mL	0.077	0.512
	Testosterone ng/dL	0.131	0.239
	Androstenedione ng/mL	0.024	0.829
	hCG mIU/mL	0.116	0.295
	FSH mIU/mL	0.148	0.182
	E2 (FF) pg/mL	0.054	0.625
Protocol 1—HMG	Progesterone ng/mL	0.244	0.174
	Testosterone ng/dL	0.026	0.879
	Androstenedione ng/mL	0.007	0.969
	hCG mIU/mL	−0.066	0.705
	FSH mIU/mL	0.257	0.139
	E2 (FF) pg/mL	0.000	1.000
Protocol 2—HMG/hCG	Progesterone ng/mL	−0.059	0.752
	Testosterone ng/dL	0.000	1.000
	Androstenedione ng/mL	0.031	0.858
	hCG mIU/mL	−0.032	0.845
	FSH mIU/mL	0.222	0.173
	E2 (FF) pg/mL	−0.085	0.603
Protocol 3—rFSH	Progesterone ng/mL	0.177	0.248
	Testosterone ng/dL	−0.086	0.537
	Androstenedione ng/mL	−0.019	0.894
	hCG mIU/mL	0.049	0.724
	FSH mIU/mL	−0.269	0.055
	E2 (FF) pg/mL	−0.105	0.453
Protocol 4—rFSH/hCG	Progesterone ng/mL	−0.012	0.944
	Testosterone ng/dL	0.305	0.069
	Androstenedione ng/mL	0.055	0.750
	hCG mIU/mL	0.253	0.132
	FSH mIU/mL	0.448	0.008
	E2 (FF) pg/mL	0.176	0.294

**Table 7:** Comparison of dCp CART levels in IVF patients using different stimulation methods.

Protocol	*n*	Mean	SD	*p*-Value
Protocol 1				
HMG	20	3.758	2.277	0.715
Allother	69	3.567	1.982	
Total	89	3.609	2.039	
Protocol 2				
HMG/hCG	22	4.424	1.922	0.030
Allother	67	3.342	2.019	
Total	89	3.609	2.039	
Protocol 3				
rFSH	27	3.356	2.170	0.443
Allother	62	3.720	1.988	
Total	89	3.609	2.039	
Protocol 4				
rFSH/hCG	20	2.908	1.468	0.080
Allother	69	3.813	2.143	
Total	89	3.609	2.039	
ProtocolMerge				
HMG/hCG	42	4.106	2.100	0.029
rFSH/hCG)	47	3.165	1.898	
Total	89	3.609	2.039	

**Table 8:** dCp CART Levels based on FSHR polymorphisms.

FSHR Polymorphism	*n*	Mean	SD	*p*-Value
FSHR				
SER/SER	32	4.198	2.257	0.098
SER/ASN	39	3.204	1.753	
ASN/ASN	11	3.149	2.260	
Total	82	3.585	2.065	
FSHR (Variant 1)				
SER/SER + SER/ASN	71	3.652	2.043	0.456
ASN/ASN	11	3.149	2.260	
Total	82	3.585	2.065	
FSHR (Variant 2)				
SER/SER	32	4.198	2.257	0.031
SER/ASN + ASN/ASN	50	3.192	1.851	
Total	82	3.585	2.065	
FSHR (Variant 2)				
SER/SER + ASN/ASN	43	3.930	2.278	0.113
SER/ASN	39	3.204	1.753	
Total	82	3.585	2.065	

## Data Availability

The original contributions presented in this study are included in the article. Further inquiries can be directed to the corresponding author.
